# Assessment of the content, occurrence, and leachability of arsenic, lead, and thallium in wastes from coal cleaning processes

**DOI:** 10.1007/s11356-018-3621-7

**Published:** 2018-11-13

**Authors:** Dorota Makowska, Andrzej Strugała, Faustyna Wierońska, Martyna Bacior

**Affiliations:** Faculty of Energy and Fuels, AGH University of Science and Technology, Mickiewicza Avenue 30, 30-059 Krakow, Poland

**Keywords:** Coal cleaning, Waste, Arsenic, Lead, Thallium

## Abstract

The aim of the study was to evaluate the content, occurrence, and leachability of arsenic (As), lead (Pb), and thallium (Tl) in wastes from coal cleaning processes with respect to the safe management of this waste. The study focused on wastes resulting from the wet gravitation and flotation processes employed for the purposes of coking coal cleaning in four coal mines situated in the Upper Silesian Coal Basin (Poland). The scope of the study included (i) determination of the content of these elements in the investigated wastes using atomic absorption spectrometry, (ii) evaluation of their mode of occurrence using electron microprobe analysis, and (iii) preliminary assessment of their leachability in deionized water. The content of the analyzed elements in the examined samples of coal waste was twice as high as the average content of these elements in the Earth’s crust. The contents of As and Pb, however, did not exceed their permissible contents in inert waste in accordance with Polish legal regulations based on EU directives. The limit on the content of Tl is not specified by these regulations, but its amount in the examined samples was similar to that occurring in the soils. Moreover, leaching tests have shown that these elements are hardly eluted from the analyzed material. Their content in the water leachates was generally lower than the detection limit of the analytical method, complying with the standards for good and very good water quality. Low leachability of these elements most probably results from their mode of occurrence in the investigated wastes. The chemical analysis using an electron microprobe and the analysis of the correlation between these elements, e.g., total and pyritic sulfur, have shown that Pb, As, and Tl are mainly found in sulfide minerals which are characterized by negligible solubility. In conclusion, the investigated hard coal processing waste does not constitute a threat to the environment and can be commercially used or safely neutralized, e.g., by landfilling.

## Introduction

Coal is one of the main fuels used in the Polish power industry and also a raw material for coke production. Raw coal (run-of-mine) is, in most cases, subjected to such operations as crushing, screening, and washing. The last of the aforementioned operations, used in order to remove gangue from raw coal, is commonly applied in the case of coking coal production to ensure coke quality parameters (coking properties and the contents of ash, sulfur, alkali, phosphorus, etc.) required by coke consumers. Coals used in Poland for combustion are not enriched to the same extent as coking coals; therefore, the content of mineral matter and sulfur is often higher (Blaschke et al. 2006). However, due to the new Best Available Techniques (BAT) Conclusions for large power plants (BAT-LCP 2017) approved by the European Commission in April 2017, which tighten up the SO_2_ emission standards and introduce restrictions on the emission of mercury (Hg) into the atmosphere, it will be necessary to improve the quality of coal used for combustion processes (Makowska et al. 2017b). Several studies (Akers and Dospoy 1994; Zhu et al. 2015; Makowska et al. 2014, 2016; Dziok et al. 2014; Zajusz-Zubek and Konieczyński 2014) have shown that the coal cleaning processes constitute an effective method for removing ecotoxic elements (such as Hg) from coal, thereby lowering the emission of these elements to the environment in the processes of combustion, gasification, and pyrolysis. On the other hand, the waste produced during coal cleaning can pose a threat to the environment because of the accumulation of ecotoxic elements mostly in this waste.

Wastes from coal mining accounted for about 26% of the total volume of wastes in Poland in 2015 (CSO 2016), of which about 94% was in the form of a tailings rock excavated with coal (Baic et al. 2011). This waste comprises waste from manual and dense medium coal cleaning, waste from jigs, flotation tailings, and sludge waste. They differ, i.a., in grain size: coarse grains come from manual and dense medium enrichment, fine wastes from enrichment in jigs, and very fine-grained wastes are produced in the process of flotation.

Management of mining wastes in the European Union, including wastes from hard coal processing, is regulated by Directive of the European Parliament (Directive 2006/21/EC, 2006) and of the European Council, which was implemented in the Polish law in 2008 (Dz.U. 2008 nr 138 pos. 865, 2008). These legal regulations indicate that, in the first place, waste production should be minimized and the generated waste should be subject to recovery. If it is not possible, the waste producer is obliged to dispose of the waste by storage and to limit its negative impact on the environment. Such a procedure allows for a reduction of the amount of produced waste, while increasing the raw material base for other industries. The utilization possibility of waste from coal cleaning processes depends on the quality of the waste, and its landfill should not have a negative impact on the environment.

Most extractive wastes undergo recovery by creating the so-called landscape constructions, i.e., the wastes are deposited on the surface of the area for reclamation or leveling, changing the landform (Mirowski and Badera 2015). Coal mining wastes can also be used in hydrotechnical and ground construction (curing sites for road construction, flood defenses, etc.), filling and backfilling of mines, for aggregate production, or, in the case of high contents of carbonaceous substances, they can be used as a fuel (coal sludge) (Góralczyk and Baic 2009; Klojzy-Karczmarczyk et al. 2016; Baic 2013a, b). However, the dumping of wastes in the form of heaps poses a lot of problems, e.g., it occupies the land, often causes destruction of the landscape, and can lead to self-ignition of the wastes and cause pollution of the terrestrial and aquatic environment (Ciesielczuk et al. 2014; Ribeiro et al. 2011; Zielinski et al. 2000). Studies (Yakun et al. 2016; Shang et al. 2016; Bhuiyan et al. 2010) indicate contamination of the soil with heavy metals as a result of coal mining. Moreover, sulfides contained in wastes in contact with air are oxidized and, together with the water seeping through the wastes, may form an effluent containing sulfuric acid, i.e., acid mine drainage. This can cause leaching of ecotoxic elements from wastes, posing a serious threat to the environment, by contaminating surface waters and aquifers (Galhardi and Bonotto 2016; Jabłońska-Czapla et al. 2015). The elevated content of harmful elements in soils and crops in areas adjacent to coal mines is a potential threat to the health of the local population (Shi et al. 2013) and may require the use of soil cleaning techniques such as biochar addition (Bielská et al. 2018). According to the Law on wastes (Dz.U. 2013 pos. 21, 2012), wastes are considered to be hazardous if elements such as, Hg, cadmium (Cd), As, Pb, and Tl exceed the defined limits (Directive 2008/98/EC, 2008). The literature on the subject of potentially ecotoxic elements in coal and coal mining wastes is very rich. Only selected reference papers closely related to the topic discussed in the article are cited here.

Hg, As, Cd, Pb, and Tl are among the most harmful elements, and their content in coal seams is usually greater than their average content in the Earth’s crust. The content of mercury in wastes from the extractive industry in Poland (including coal processing wastes) and its environmental impact have recently been the subject of numerous papers (Dziok et al. 2015; Michalska and Białecka 2012; Wichliński et al. 2016). A number of studies have also been carried out for the existing coal waste heaps in terms of Hg content and leaching properties (Klojzy-Karczmarczyk and Mazurk 2010, 2014). There are many studies in the literature focusing on ecotoxic elements in landfills of mining wastes (heaps, excavation filling, land leveling, etc.) and in wastes from coal conversion processes such as fly ash or slag (Skodras et al. 2006, 2009; Grammelis et al. 2006; Shin et al. 2013; Tsiridis et al. 2015; Phoungthong et al. 2018; Wierońska et al. 2017, 2018; Styszko et al. 2018). However, less information is available in the literature on the content of ecotoxic elements in “fresh” wastes (i.e., wastes that have not been exposed to environmental factors which occur during their storage due to the short period of time since their production) derived from mechanical processing of hard coal and on their impact on the environment. Such data is necessary to classify mining wastes as inert wastes and would allow for its commercial use, for example, in civil constructions. Moreover, it is necessary to update and monitor such data, due to the high variability of the content of these elements in mining wastes, resulting from the variability of coal seams and general instability of the quality of wastes from enrichment processes (Baic and Blaschke 2011). This research also allowed for assessing the suitability of coal enrichment processes as precombustion methods for limiting the emissions of harmful substances from coal conversion processes.

The aim of the presented research was to evaluate the environmental hazards resulting from the use and/or disposal of wastes from coal cleaning processes, in terms of the content of some of the most harmful ecotoxic elements. The scope of the study included analyses of the content of selected elements, i.e., As, Pb, and Tl, in wastes generated during coal gravitation enrichment and in the flotation tailings. As for other potentially toxic elements, a reliable assessment of the Cd content in the studied material was not possible because its concentration in the investigated samples was below the detection limit of the analytical method employed. Hg content in these wastes has already been investigated in a previous study (Dziok et al. 2015). Investigations of the mode of occurrence (i.e., mineral forms and mineral associations) of the selected elements in the coal wastes and their leaching behavior in deionized water were also conducted.

## Materials and methods

### Examined material

The study focused on wastes from the enrichment processes of coking coal, coming from four mines situated in the Upper Silesian Coal Basin, Poland. Samples of waste from various coal cleaning operations, i.e., dense medium washery, jigs, and flotation, were examined. The examined samples were collected, prepared, homogenized, and stored by a certified laboratory according to the ISO 13909:2016 standards. In order to ensure adequate representativeness of samples, the primary samples of each waste material collected daily over a period of 1 month were combined into one sample of each waste which was subsequently homogenized and reduced for analyses. General characteristics of the samples are shown in Table 1. The total number of the examined samples was 12.

**Table 1:** Characteristics of the examined samples of wastes from coal cleaning processes

No.	Mine	Enrichment process	*M*_*t*_^*ar*^ [%]	*M*^*a*^ [%]	*A*^*a*^ [%]	*S*_*t*_^*a*^ [%]	*S*_*p*_^*a*^ [%]	*C*^*a*^ [%]	*Fe*^*a*^ [%]
1.	A	Dense medium washery	1.4	0.9	79.4	0.26	0.24	11.2	4.3
2.		Jigs	2.9	0.8	81.5	0.22	0.2	10.7	3.0
3.		Flotation	20.8	1.0	71.7	0.50	0.48	17.7	3.0
4.	B	Dense medium washery	0.9	0.9	83.8	0.12	0.10	9.1	2.6
5.		Jigs	2.3	0.9	86.1	0.13	0.11	6.9	2.5
6.		Flotation	13.6	1.2	50.9	0.37	0.28	40.0	2.6
7.	C	Dense medium washery	1.8	1.8	77.8	1.04	0.85	11.9	3.8
8.		Jigs	5.4	1.7	79.2	0.33	0.32	11.2	3.0
9.		Flotation	21.0	1.9	73.1	0.25	0.21	15.2	2.2
10.	D	Dense medium washery	1.6	0.9	72.6	0.16	0.15	18.0	3.4
11.		Jigs	3.3	0.8	79.7	0.29	0.26	11.3	4.7
12.		Flotation	19.2	1.0	65.8	0.28	0.24	23.6	2.2

*M*_*t*_ total moisture content; *M* moisture content; *A* ash content; *S*_*t*_ total sulfur content; *S*_*p*_ pyritic sulfur content; *C* carbon content; *Fe* iron content; ^*ar*^ as received; ^*a*^ in analytical sample

**Fig. 1:**
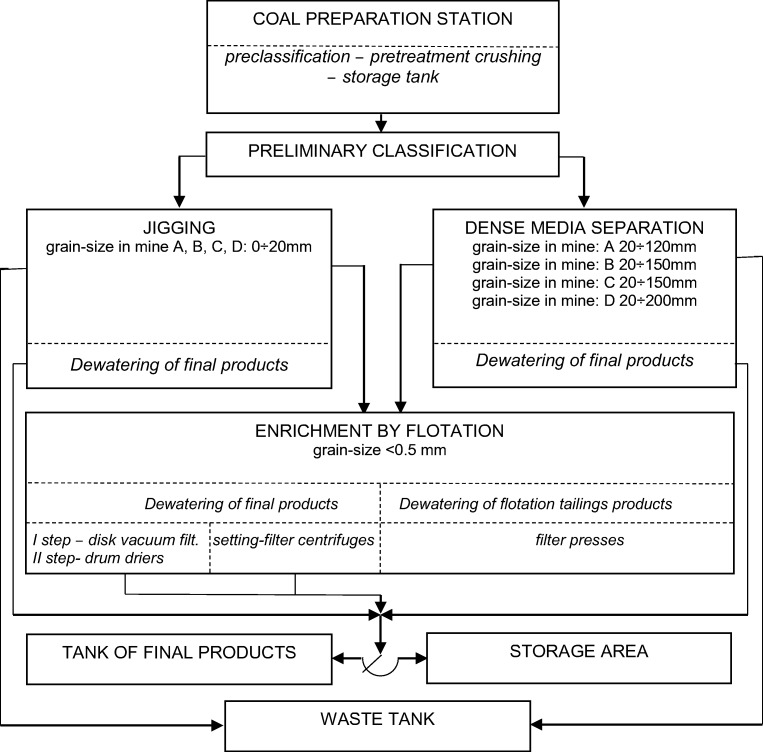
General scheme of coal cleaning in the analyzed mines

The coal cleaning process in mines A–D was carried out according to the general scheme shown in Fig. 1. The grain size of the examined coal wastes was different: the waste from the dense medium washery had grains with a diameter over 20 mm; the waste from jigs, below 20 mm; and the waste from the flotation process had a grain size smaller than 0.5 mm.

### Analytical methods

The analyses of the contents of moisture, ash, total sulfur, pyritic sulfur, and carbon were carried out in accordance with PN-ISO: 1171:2002, 571:2002, 351:1999, as well as PN-G-: 04571:1998 and 04582:1997P.

The determinations of the contents of As, Pb, Tl, and iron (Fe) were performed by means of atomic absorption spectrometry using a Hitachi Z-2000 Spectrometer. The determinations of As and Tl contents in the samples were performed using electrothermal atomization in a graphite furnace, whereas Pb and Fe contents were determined through flame atomization. For the determinations of Tl and Pb contents, the analytical samples of the wastes were subjected to slow combustion at 600 °C and then digested in a microwave system SpeedWave 4 Berghof using concentrated nitric acid (V) (EMSURE ACS, Reag. Ph Eur). Determinations of As and Fe contents were performed for the analytical samples also by digestion in a microwave system but in a mixture of concentrated nitric acid (V) (EMSURE ACS, Reag. Ph Eur) and hydrofluoric acid (Baker Instra-Analyzed, for Trace Metal Analysis), using a supersaturated solution of boric acid (Acros Organics, extra pure–trace metal basis) as a complexing reagent.

In order to pre-assess the mode of occurrence of the analyzed elements, a statistical analysis of the correlation between their content and the content of ash, total sulfur, pyritic sulfur, and the organic matter (as C element content) was carried out. For these purposes, the regression analysis by means of F-Snedecor test was used with the application of the Statistica software.

A JEOL JXA-8230 Electron Probe Microanalyzer (EPMA) was used to determine the modes of occurrence of As, Pb, and Tl (i.e., to identify their forms of occurrence). The analysis of the chemical composition allowed for the identification of the mineral components and their accompanying trace elements. Samples for the analysis were prepared in the form of cuts with a diameter of 1 in. For this purpose, representative sample grains were embedded in an epoxy resin and polished using diamond abrasives. In order to ensure proper conductivity, the samples prepared in such a way required additionally coating their surface with the graphite layer before making the measurements. The measurements were carried out under the following operating conditions: acceleration voltage of 20 kV (for sulfides) and 15 kV (for aluminosilicate minerals, oxides, and carbonates), probe current of 20 nA, and the spot size 1–5 μm. The analyzed elements (As, Pb, Tl) were determined with the use of the L-type X-ray spectrometer equipped with pentaerythritol crystal (PETL), the H-type X-ray spectrometer equipped also with pentaerythritol crystal (PETH) or thallium acid phthalate crystal (TAPH), and J-type X-ray spectrometer with pentaerythritol crystal (PETJ). For the measurements of these elements in sulfide minerals, the following analytical lines, crystals, and standards were used: As (Lα, TAPH, GaAs); Pb (Mα, PETJ, galena); Tl (Mα, PETL, TlBrI). While for the measurements of these elements in aluminosilicate minerals, oxides, and carbonate minerals, the following parameters were used: As (Lα, TAPH, InAs); Pb (Mα, PETH, crocoite); Tl (Mα, PETL, TlBrI).

A preliminary leaching test was carried out for samples from one mine, involving the preparation of water leachates in accordance with EN 12457-4:2006, by shaking the wastes in deionized water at a mass ratio of 1:10 at ambient temperature for 24 h (basic test). The contents of the analyzed elements in the leachates were determined by means of atomic absorption spectrometry with atomization in a graphite furnace based on the standard PN-EN ISO 15586:2005. The application of this leachability test results from the European legal regulations (Decision 2003/33/EC, 2002) introduced into the Polish law by the Act (Dz.U. 2015 pos. 1277, 2015). This test simulates the conditions of waste storage, where the waste and the substances leached out of it determine the pH of the water flowing through it or draining from it.

Indicators such as the enrichment factor (*EF*) and the geo-accumulation index (*I*_geo_) were used in order to evaluate the enrichment rate of As, Pb, and Tl in the examined wastes. The *EF* factor was estimated according to formula (1) (Bhuiyan et al. 2010) with reference to Fe as the normalizing element, the average content of which in the Earth’s crust is about 5% (Clarke and Washington 1924). The geochemical background was the average content of the element in the upper part of the Earth’s crust.

1 $$ EF=\frac{\frac{C_n}{C_{Fe}}}{\frac{B_n}{B_{Fe}}} $$

where:*C*_*n*_, content of analyzed element in the tested material; *C*_*Fe*_, content of normalizing element (Fe) in the tested material; *B*_*n*_, geochemical background of the analyzed element; *B*_*Fe*_, geochemical background of the normalizing element (Fe).

The geo-accumulation index *I*_geo_ is one of the indicators describing the degree of pollution of, e.g., a soil or sediment (Baran and Wieczorek 2015; Yakun et al. 2016; Bhuiyan et al. 2010; He et al. 2015) and was introduced by Müller (1969). This index was estimated according to formula (2), taking as the geochemical background the average content of the analyzed elements in the Earth’s crust.

2 $$ {I}_{geo}={\log}_2\left(\frac{C_n}{1.5\bullet {B}_n}\right) $$

where:*C*_*n*_, the analyzed element in the tested material; *B*_*n*_, geochemical background of the analyzed element

The level of pollution is determined by the value of this indicator: 0, no pollution *I*_geo_ < 0; 1, slight pollution 0 < *I*_geo_ ≤ 1; 2, slightly moderate pollution 1 < *I*_geo_ ≤ 2; 3, moderate pollution 2 < *I*_geo_ ≤ 3; 4, slightly high pollution 3 < *I*_geo_ ≤ 4; 5, high pollution 4 < *I*_geo_ ≤ 5; 6, very high pollution *I*_geo_ > 5.

## Results and discussion

### Content of As, Pb, and Tl in wastes from coal cleaning process

The As content in the examined wastes varied from 5.5 to 23.3 mg/kg db (dry basis), and its average content was 11.5 mg/kg db. The average crustal content of As is about 1.8 mg/kg (Kabata-Pendias 2011), so the mean value of the *EF* in the examined wastes was 10.3.

**Fig. 2:**
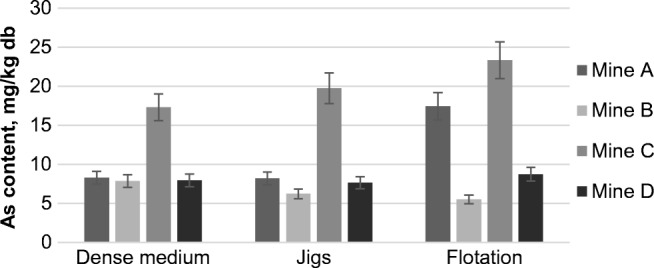
Arsenic content in the wastes from the coal cleaning processes

Based on the results presented in Fig. 2, no conclusions can be drawn about the influence of different types of cleaning operations on the content of As in the examined samples. However, it can be seen that wastes from the gravity cleaning methods (dense medium and jigs) were characterized by a smaller variability of the As content than the wastes from the flotation process. The content of As in the examined wastes also differed from mine to mine. The highest content of As was in the wastes coming from mine C, for which the average content was 20.1 mg/kg db. Samples from mines B and D contained a similar amount of As (6.5 and 8.1 mg/kg db, respectively) and for mine A, a slightly higher amount was determined (11.3 mg/kg db on average). This suggests that the content of this element in the wastes from coal cleaning can be strongly influenced by the mode of As occurrence in the samples, due to the nature of coal.

**Fig. 3:**
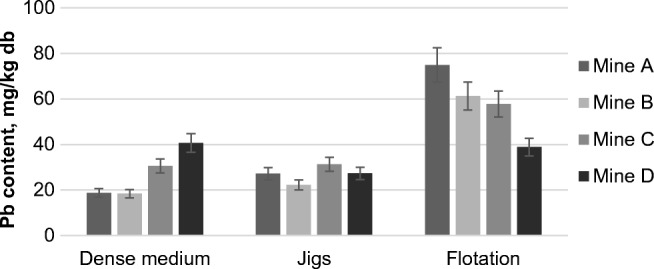
Lead content in the wastes from the coal cleaning processes

The Pb content in the examined samples also exceeded its mean content in the upper continental crust which is equal to about 17 mg/kg of crust (Rudnick and Goa 2003). The average Pb enrichment factor *EF* for the wastes was approximately 3.9. From the comparison shown in Fig. 3, it can be concluded that the type of the enrichment process had a greater influence on the content of this element than the nature of coal itself (mine). The largest amount of Pb was recorded in flotation tailings, for which the average value was 58 mg/kg db. A lower content of this element was registered for the samples from gravity enrichment (average content of 27 mg Pb/kg of waste db).

**Fig. 4:**
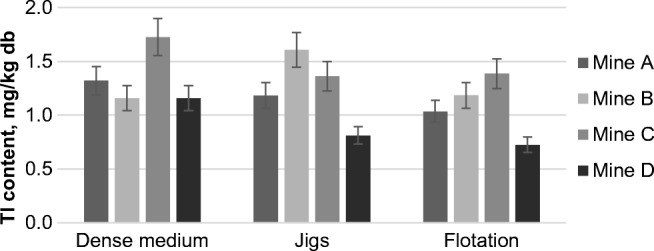
Thallium content in the wastes from the coal cleaning processes

The amount of Tl in the examined wastes was similar (0.7–1.7 mg/kg db) for all samples. The average content of Tl in the upper parts of the Earth’s crust is about 0.55 mg/kg (Hu and Gao 2008), while the average content in the samples was 1.2 mg/kg. The average enrichment factor *EF* is 3.8 (similar to lead *EF*). Based on Fig. 4, it is difficult to draw conclusions concerning the factors influencing the content of this element in the wastes from coal cleaning.

The Regulation of the Polish Ministry of Environment on the criteria for the classification of extractive wastes concerning the inert wastes (Dz.U. 2011 nr 175 pos.1048, 2011) based on the EU regulation (Directive 2006/21/EC, 2006) states that extractive wastes may be considered inert if, i.a., the contents of substances potentially hazardous to the environment or the health and life of humans do not exceed the soil or ground quality standards defined for group B soils. Indeed, As and Pb are categorized as potentially dangerous substances for which limit values are defined. Group B refers to agricultural soils, forests, built-up and urbanized areas (excluding industrial areas), barren and fossil lands, and road transport infrastructures (Dz.U. 2002 nr 165 pos.1359, 2002—act canceled), which corresponds to group I soils according to the law currently in force (Dz.U. 2016 pos.1395, 2016).

The average contents of both As and Pb did not exceed the permissible values for soils from group I (Table 2). Therefore, these wastes may be regarded as inert wastes. Only four samples contained slightly more As than the lowest acceptable value for some soils from group II (agricultural areas, national parks, and nature reserves). No sample exceeded the allowable content of Pb for other groups of soils.

**Table 2:** Average, minimum, and maximum contents of As, Pb, and Tl, and their permissible concentrations in soils or ground (Dz.U. 2016 pos.1395, 2016)

Element	Content in wastes [mg/kg db]		Allowable concentration in soil or ground [mg/kg db]					
			Group I	Group II			Group III	Group IV
				II-1	II-2	II-3		
As	Average	11.5	25	10	20	50	50	100
	Min.	5.5						
	Max.	23.3						
	Number of wastes exceeding the allowable content		0	4	1	0	0	0
Pb	Average	37.4	200	100	250	500	500	600
	Min.	18.4						
	Max.	74.9						
	Number of wastes exceeding the allowable content		0	0	0	0	0	0
Tl	Average	1.2	Not specified					
	Min.	0.7						
	Max.	1.7						

Both Polish and EU regulations do not specify the allowable content of Tl in soils or ground. One of the few countries where soil and sediment quality standards were specified as regards the acceptable levels of this element is Canada (CME 2011). The permissible content of Tl for soils in Canada is 1 mg/kg db. According to Kabat-Pendias and Pendias (1993), the content of Tl in uncontaminated soils ranges from 0.02 to 2.8 mg/kg, but a content above 1 mg/kg may indicate anthropogenic origin. On this basis, it can be concluded that the average content of this element in the examined samples does not significantly differ from its normal content in soils. For comparison, the content of Tl in soils contaminated with this element was reported as ranging from 4.42 to 49.82 mg/kg (Woch et al. 2013).

The values of the geo-accumulation index *I*_geo_ and the levels of contamination for the examined wastes determined on this basis are shown in Table 3. The contamination levels of the samples for As, Pb, and Tl were the same, i.e., within the range of 0 to 2, which means that the wastes were, at most, moderately polluted with these ecotoxic elements.

**Table 3:** Geoaccumulation indices *I*_geo_ and levels of contamination for the examined wastes

Value	As		Pb		Tl	
	*I*_geo_	Level	*I*_geo_	Level	*I*_geo_	Level
Average	0.26	1	0.41	1	0.53	1
Min.	− 0.63	0	− 0.47	0	− 0.19	0
Max.	1.45	2	1.55	2	1.07	2
SD	0.71	–	0.66	–	0.36	–

### Modes of occurrence of As, Pb, and Tl in the examined wastes

The impact of the ecotoxic elements contained in wastes on the environment depends on the mode of their occurrence in these wastes (Bourg 1988). The correlations between the content of these elements and the ash content as well as the selected coal components (carbon, total sulfur, and pyritic sulfur) were examined to assess the mode in which the analyzed elements occurred in the wastes. For this purpose, the Pearson’s correlation coefficient was calculated for the analyzed relationships and their statistical significance was verified (Springer 2006). The results are given in Table 4.

**Table 4:** Pearson *R* correlation coefficients for the relationship between Pb, As, and Tl and other components of the examined wastes, i.e., ash, carbon, total sulfur, and pyritic sulfur

Waste component	Pb^d^ [mg/kg]	As^d^ [mg/kg]	Tl^d^ [mg/kg]
Pb^d^ [mg/kg]	–	–	–
As^d^ [mg/kg]	0.408	–	–
Tl^d^ [mg/kg]	− 0.203	0.325	–
A^d^ [%]	− 0.696*	0.094	0.346
S_t_^d^ [%]	0.202	0.438**	0.380
S_p_^d^ [%]	0.224	0.482**	0.351
C^d^ [%]	0.646*	− 0.178	− 0.327

*Significant correlation for *α* = 0.05; **significant correlation for *α* = 0.15

^d^On dry basis

**Fig. 5:**
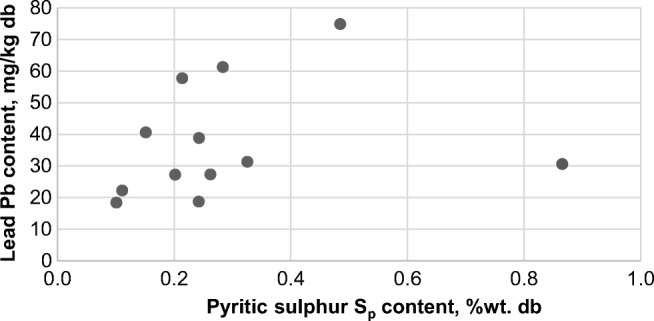
Correlation between lead content and pyritic sulfur in the examined wastes

The results of the statistical analysis show that the Pb content is directly proportional to the carbon content and inversely proportional to the ash content. This indicates the association of Pb with organic matter or its occurrence in the form of carbonates. Despite the lack of a statistically significant correlation between the Pb content and the total and pyritic sulfur content for the whole sample population, there is a tendency of the Pb content to increase in the samples as the pyritic sulfur content rises (Fig. 5). On this basis, it can be assumed that Pb in the wastes is present in the form of sulfides, which is confirmed by previous studies (Makowska et al. 2016). Both sulfides and lead carbonates are insoluble in water.

The contents of As and Tl have no statistically significant correlation with the contents of ash, total sulfur, and pyritic sulfur, and the content of carbon (*α* = 0.05) (Table 4). Therefore, it is not possible, at least from this data, to draw conclusions about the form in which these elements occur in the examined wastes. However, there is a tendency of As and Tl content to increase with the rising content of total and pyritic sulfur. In the case of As content, this tendency is confirmed by a statistically significant correlation between As content and both total and pyritic sulfur contents for *α* = 0.15. The connection between the Tl content and the content of ash, total sulfur, and pyritic sulfur was already verified in a study carried out for raw coals and their enrichment products (Makowska et al. 2017a). It should be emphasized that the association of As and Tl with sulfides in the examined wastes can be, at most, merely probable. This probability is confirmed by other studies (Yudovich and Ketris 2005; López Antón et al. 2013). The negative correlation coefficients between the As as well as the Tl content and the content of C indicate the lack of their association with organic matter. Sulfides, which may contain As or Tl in their crystalline structure, are also water-insoluble minerals.

**Fig. 6:**
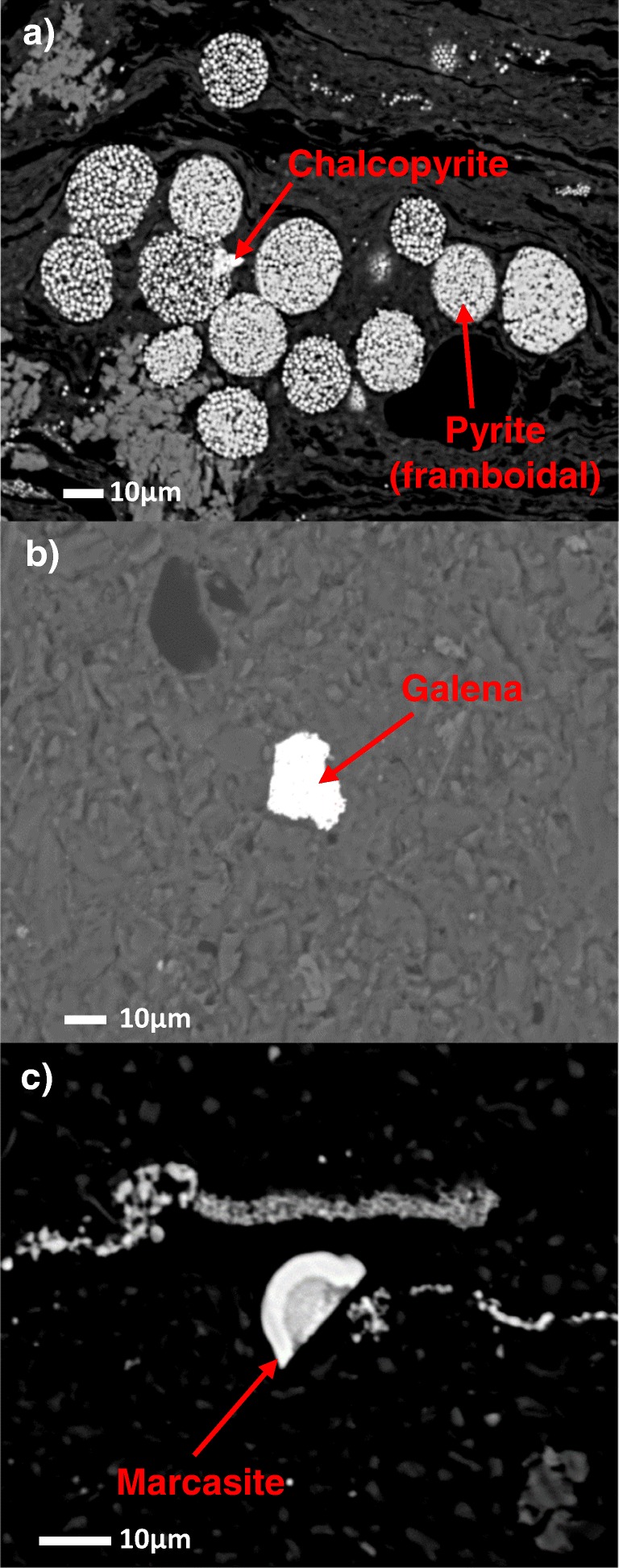
Images from the electron microscope analysis of wastes from mines C and D: **a** framboidal pyrite with fused chalcopyrite; **b** galena in granite or postgranite sandstone grain; **c** marcasite

The microscopic analysis in combination with the point analysis of the chemical composition of the wastes from C and D mines was carried out by means of EPMA in order to accurately determine the modes of occurrence of Pb, As, and Tl. Examples of micrographs of the observed mineral phases are shown in Fig. 6.

This analysis confirmed the association of Pb with sulfides (Table 5). Lead occurred mainly in the form of galena (PbS) and, in small amounts, in pyrite (FeS_2_), marcasite (FeS_2_), and chalcopyrite (CuFeS_2_). Also, a small amount of it was recorded in carbonates. In the case of both As and Tl, their presence in the form of their minerals (e.g., arsenopyrite (FeAsS), realgar (AsS), auripigment (As_2_S_3_), lorandite (TlAsS_2_), or hutchinsonite ((Tl,Pb)_2_As_5_S_9_)) in the investigated sample cuts was not observed, while both of them were found in sulfide minerals (Table 5): Tl in marcasite (FeS_2_), galena (PbS), chalcopyrite (CuFeS_2_), and pyrite (FeS_2_), while As in marcasite (FeS_2_) and pyrite (FeS_2_).

**Table 5:** Chemical composition of the mineral phases contained in the wastes from mines C and D

Mineral	Chemical formula	Number of measurements, *n*	Content [%wt.]		
			Pb	As	Tl
Galena	PbS	3	81.2–87.6	0	0–0.209
Chalcopyrite	CuFeS_2_	4	0.077–0.131	0–0.084	0.071–0.165
Pyrite	FeS_2_	3	0–0.227	0–1.321	0.056–0.093
Marcasite	FeS_2_	2	0.248–0.537	1.245–1.346	0.158–0.213
Siderite	FeCO_3_	5	0–0.025	0	0

### Assessment of the leaching properties of Pb, As, and Tl from the wastes from coal cleaning processes

The results of the leaching experiments for As, Pb, and Tl from three selected samples are shown in Table 6. The contents of these elements in the aqueous leachates in most cases were below the detection limit (DL) of the analytical method. Only in the water extract from the waste from the enrichment in the jigs was the Pb content detectable, but this value did not exceed the hydrochemical background for groundwater (Dz.U. 2015 pos. 85, 2015). In the case of all analyzed elements (As, Pb, Tl), it can be stated that the water leachates met the requirements for class I water.

**Table 6:** As, Pb, and Tl contents in water leachates and the allowable values of these elements in groundwater (Dz.U. 2015 pos. 85, 2015)

Element	Unit	Sample	Content in leachate	Standard deviation (SD)	Hydrochemical background	Allowable content for water class				
						I	II	III	IV	V
As	mg/dm^3^	Wastes from dense medium washery	< 0.0014*	0.0008	0.00005–0.02	0.01	0.01	0.02	0.2	> 0.2
		Wastes from jigs		0.0008						
		Wastes from flotation		0.0004						
Pb	mg/dm^3^	Wastes from dense medium washery	< 0.0019*	0.0014	0.001–0.01	0.01	0.025	0.1	0.1	> 0.1
		Wastes from jigs	0.0031	0.0006						
		Wastes from flotation	< 0.0019*	0.0015						
Tl	mg/dm^3^	Wastes from dense medium washery	< 0.0001*	0.0000	0–0.00001	0.001	0.01	0.02	0.1	> 0.1
		Wastes from jigs		0.0002						
		Wastes from flotation		0.0001						

*The content of the element is below the detection limit (DL) of the analytical method

The contents of As and Pb in the examined extracts did not exceed the allowable values for leachates from inert waste defined for landfill, that is 0.5 mg/dm^3^ for both As and Pb in accordance with the EU law (Decision 2003/33/EC, 2002). Moreover, the contents of these elements did not exceed the allowable values of surface water quality indicators used while supplying the population with drinking water (allowable values: 0.001 mg As/dm^3^, 0.005 mg Pb/dm^3^, Tl not specified) (Dz.U. 2002 nr 204 pos. 1728, 2002). The water leachates also met the criteria for As and Pb contents (the criterion for Tl has not been specified) in drinking water provided by the World Health Organization and EU legislation (MCL maximum contaminant level for As and Pb is 0.010 mg/dm^3^) (WHO 2011; Directive 98/83/EC, 1998).

The amount of lead leached from the wastes from jigs, calculated in relation to the dry mass of the material, was equal to 0.03 mg/kg on average. This value is significantly lower than the permissible leachability of lead from inert wastes (for basic test on 1 kg of material: 10 dm^3^ of elution liquid) which is equal to 0.5 mg Pb/kg db (Dz.U. 2015 pos. 1277, 2015; Dz.U. 2013 pos. 38, 2013).

In the case of Tl, the only requirements for its allowable content in water are given in The Regulation of the Polish Ministry of Environment on the conditions for the introduction of sewage into waters (Dz.U. 2014 pos. 1800, 2014) (established according to the EU law Directive 91/271/EEC 1991, and Directives 2010/75/EU 2010). Thallium is classified (along with As and Pb) under substances particularly harmful to the aquatic environment, causing water pollution that should be limited. Its permissible content in wastewater should not exceed 1 mg/dm^3^ and in the case of the analyzed water leachates the content of Tl did not exceed 0.0001 mg/dm^3^.

However, it should be clearly stated that the preliminary leaching test of the ecotoxic elements from the examined wastes may not give a full picture of the leaching behavior of these elements. Comprehensive research including the effect of, e.g., the pH of the solution, the time of contact of the material with the washing solution, the ratio of the amount of material to the amount of solution, the grain size of the material, etc., on the leaching behavior of these elements should be carried out in order to draw final conclusions.

## Conclusions

The results of the study of the contents of As, Pb, and Tl in wastes from coal cleaning processes indicate that this waste should not pose a threat to the environment, despite the fact that the amount of these elements is higher when compared to their mean content in the upper Earth’s crust. According to Polish and EU regulations, the examined wastes could be categorized as inert wastes and potential raw materials for earthworks or land reclamation. However, final conclusions in this case require additional studies involving a larger number of samples.

Based on the tests of As, Pb, and Tl leaching from the examined wastes, it can be concluded that these elements are practically not subject to washout. Indeed, their content in water leachates did not exceed the allowable values for water class I. This is probably due to the presence of the elements in water-insoluble forms such as sulfides and carbonates. Based on the analysis of the occurrence of Pb, As, and Tl in the samples and their leaching, it can be concluded that these wastes do not pose a threat to the environment and, thus, they can be used commercially or safely landfilled. However, it should be noted that the leaching of ecotoxic elements is a complex process, and the degree to which they dissolve in the leaching liquid will depend not only on the material properties (including the form of their occurrence in the tested material) but also on environmental conditions and on the time of material contact with the liquid. Therefore, it is necessary to conduct a broader study taking into account, for example, the scenario of co-deposition of these wastes with municipal wastes (application of the TCLP test), in order to obtain a full picture of the possible impact of the investigated wastes on the environment.
